# Prospective evaluation of pain control and quality of life in patients with chronic pancreatitis following bilateral thoracoscopic splanchnicectomy

**DOI:** 10.1007/s00464-013-2937-0

**Published:** 2013-04-10

**Authors:** Malgorzata B. Malec-Milewska, Wieslaw Tarnowski, Adam E. Ciesielski, Emilia Michalik, Maciej R. Guc, Jacek A. Jastrzebski

**Affiliations:** 1 Department of Anesthesiology and Intensive Care, Medical Center for Postgraduate Education, Ul. Czerniakowska 231, 00-416 Warsaw, Poland; 2 Department of General and Digestive Tract Surgery, Medical Center for Postgraduate Education, Warsaw, Poland; 3 Institute for Biocybernetics and Biomedical Engineering, Polish Academy of Sciences, Warsaw, Poland

**Keywords:** Chronic pancreatitis, Pain management, Bilateral thoracoscopic splanchnicectomy, Quality of life

## Abstract

**Background:**

Abdominal pain in chronic pancreatitis (CP) is the most common symptom with a highly unfavorable impact on the quality of life. It has been shown that bilateral thoracoscopic splanchnicectomy (BTS) may produce marked pain relief for the majority of patients. The aim of this study was to evaluate the effectiveness of BTS in pain control and quality-of-life improvement in patients with a severe form of CP.

**Methods:**

Between April 2000 and April 2009, a total of 30 patients qualified for BTS due to CP-related pain. Their age ranged from 28 to 60 years. A 12-month follow-up period was planned for all the patients enrolled. To evaluate effectiveness of BTS, an 11-point Numeric Rating Scale (NRS) and the Quality of Life Questionnaire C-30 (QLQ-C30) in its basic form, developed by European Organization for Research and Treatment of Cancer, were used. An NRS value between 0 and 3 was considered a positive postoperative pain control result.

**Results:**

The bilateral splanchnicectomy procedure was performed successfully in 27 of 30 qualified patients. A positive effect based on decreased pain (*p* < 0.05) at 12 months was achieved in 24 patients (80 %). The initial change in quality of life was not significant but it gradually improved with time (preop vs. 12 months QLQ-C30 score, *p* < 0.001).

**Conclusions:**

This study showed that BTS is safe and efficacious for pain alleviation in patients with severe CP. It may significantly increase the chances of a long-lasting, life-changing improvement in the quality of life.

Chronic pancreatitis (CP) is a long-lasting inflammatory process responsible for irreversible degeneration of pancreatic tissue, which is gradually replaced with fibrous tissue [1]. CP risk factors include increased alcohol use, smoking, autoimmune factors obstructing secretion of pancreatic juice, and genetic predisposition [2, 3]. It is currently acknowledged that CP-related pain has a multifactorial etiology, while until quite recently its sole cause was presumed to be increased pressure in pancreatic ducts and subsequent ischemia, which lead to release of free radicals and stimulation of specific nociceptors and/or celiac plexus which innervates the pancreas. Normal pancreatic duct pressure is 7–15 mmHg and in CP it may increase to 80 mmHg. The above theory is supported by the results of clinical studies in which a significant decrease in pain intensity was noted once pancreatic duct pressure was lowered and/or antioxidant therapy was introduced [4–7]. Some authors emphasize that the pathogenesis of pain in CP is strictly linked to inflammation within the pancreatic duct wall, its destruction, and, effectively, pancreatic autodigestion. Other pain-potentiating factors include episodes of acute pancreatitis, bowel motility disorders, intrapancreatic neuropathic changes, and local abnormalities such as biliary system obstruction, duodenal narrowing, pseudocysts, or pancreatic cancer. Pain affects 75–90 % of CP patients. It is often accompanied by other gastrointestinal symptoms: flatulence, fullness, nausea, vomiting, and diarrhea [1]. In many [8] but not all cases [9], in the advanced stages of the disease, pain may subside. Poor pain control means not only a poor quality of life but also a decreased ability to pursue professional development, leading to social disability [10].

With the exception of autoimmune and some obstructive forms of the disease, there is no causative treatment for CP. Its symptomatic management is based on pain control, pancreatic enzyme supplements, compensation of carbohydrate metabolism disturbances, and prevention of malnutrition. A vital part of the treatment process is abstinence from alcohol and smoking cessation, as well as obeying strict dietary rules. As pain management in CP has proven to be challenging, invasive methods like celiac plexus neurolysis and surgical denervation of the pancreas have been proposed for cases where resection and drainage procedures are not indicated or when their benefit-to-risk ratio has deemed them not plausible [11]. Unilateral or bilateral thoracoscopic transection of the splanchnic nerves has been used for a number of years now, with variable reported efficacy and surgical complication rate, providing immediate pain relief in up to 80 % of CP patients, with the average long-term improvement rate being much less, at about 50 % [12–14].

In this study we aimed to assess the efficacy of bilateral thoracoscopic splanchnicectomy in providing pain relief and quality-of-life (QOL) improvement in the immediate postoperative period and during 1 year of follow-up in patients with severe chronic pancreatitis.

## Materials and methods

The study protocol was reviewed and approved by the Institutional Ethics Committee. Of the 52 consecutive patients hospitalized between April 2000 and April 2009 in the Clinic of General and Digestive Tract Surgery, Medical Center for Postgraduate Education in Warsaw due to the symptoms of CP, 30 underwent the bilateral thoracoscopic splanchnicectomy (BTS) procedure. The diagnosis of CP was based on the clinical assessment (typical symptoms and medical history, the course of the disease), as well as the results of the imaging studies [endoscopic ultrasound, computed tomography, and/or endoscopic retrograde cholangiopancreatography (ERCP)]. All patients met the specific inclusion criteria: diagnosed small-duct CP, poor or no response to noninvasive treatment, average pain intensity >7 points in NRS, and sufficient mental capacity to fill out the Quality of Life Questionnaire. They all agreed to participate in the study and signed the informed consent form. Patients with large pancreatic cysts, significantly distended pancreatic duct (when drainage procedures are recommended), diagnosed massive adhesions in the pleural cavity, and intellectual disability were excluded from the study. Following the procedure, a follow-up period was set at 12 months for all the patients included in the study; it was based on the prescheduled outpatient visits in the pain clinic. Three of the patients had been unsuccessfully treated with neurolysis of the celiac plexus in another center. Pain intensity was measured using the Numeric Rating Scale (NRS), which ranged from 0 to 10, where 0 was no pain and 10 was the worst pain imaginable. Quality of life was assessed using the Quality of Life Questionnaire version 30 (QLQ-C30) of the European Organisation for Research and Treatment of Cancer (EORTC) [15]. The original self-reporting QLQ-C30 questionnaire was adapted for the Polish language by Polish researchers [16]. The questionnaire consists of 30 questions, of which 24 form nine multiquestion scales: one global scale, five functional scales (physical, role, emotional, cognitive, and social) and three symptoms scales (pain, nausea, and fatigue). The six remaining questions aim at reporting six symptoms: dyspnea, insomnia, appetite, constipation, diarrhea, and financial difficulties. The QLQ-C30 produces a complex assessment of the health-related quality of life (HR-QOL). Results are linearly recalculated to a uniform scale of 0–100 points, in which a higher score indicates a more favorable outcome. Although originally designed for cancer patients, the QLQ-C30 was previously shown to serve well as an assessment tool for the CP population [7, 17–19]. NRS and QLQ-C30 were filled out by the patients the day before the surgery and 24 h after surgery, then at 3, 6, 9, and 12 months after the procedure. Success in pain reduction was defined as the drop in NRS score to the values of 0–3 postoperatively and at 12 months.

All the patients were informed about the purpose and the protocol of the study. How to use the NRS and fill out the QLQ-C30 questionnaire was thoroughly explained to them. Cronbach’s α was used to test the reliability of the answers. Cronbach’s α is a coefficient of internal consistency that is calculated using three variables: number of test items (questions), interitem variance, and variance of the sum of answers. The value of 0.7 or above is indicative of good reliability. Statistical analysis used to establish the significance of changes in pain intensity and QOL was based on the *t* test or the Mann–Whitney *U* test, depending on the difference of variances of the tested samples. The Kolmogorov–Smirnov test was used to test for normality. To investigate the relationship between NRS and QLQ-C30 results, Pearson’s correlation was calculated. For all data analysis, *p* < 0.05 was considered statistically significant.

### Surgical technique

The surgical procedure used was based on the technique described by Cuschieri et al. [20]. After completion of the procedure, patients were always monitored for 24 h in the ICU and then discharged [21].

## Results

**Fig. 1:**
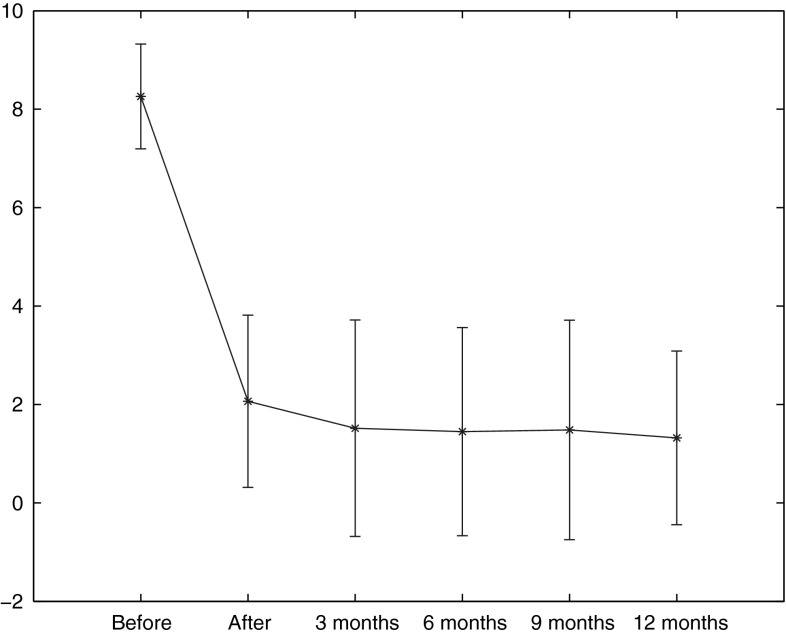
Mean values and standard deviations of the NRS score before and after BTS

Bilateral splanchnicectomy was successfully performed in 27 of the 30 patients who qualified for the procedure; hence, the statistical analysis was done on data derived from 27 cases. The demographics of the study group are given in Table 1. In two patients, massive adhesions within the pleural cavity made deflation of the lungs impossible and the procedure was waived. In these patients celiac plexus neurolysis was performed and NRS/QOL assessment continued for 12 months. Both patients reported reduction in pain intensity from 8 to 5 points in NRS. The reduction of pain by 3 points in both patients was sustained for the whole 12 months of follow-up. In one of the patients, only a right splanchnicectomy was performed due to pleural adhesions on the left side. In this case initial reduction in pain to NRS 3 was noted, which subsequently increased, reaching 5 at 12 months. The above three cases resulted in a more meticulous approach to preoperative assessment. Subsequent closer scrutiny in reviewing preoperative chest X-rays has helped us avoid further technical failures. In one case the damage to the intercostal artery on the left side occurred, which required conversion to open surgery, and splanchnicectomy on the opposite side was postponed. The right-sided thoracoscopic procedure was performed 5 months later, at which time the 12-month follow-up was started, with success in pain reduction at 1 year (NRS 0–3). In only one case, the left-sided pneumothorax was found postoperatively, which required no intervention and resolved spontaneously. One patient died 3 months after the procedure. A postmortem examination revealed the presence of pancreatic cancer (adenocarcinoma). Contact with one patient was lost after the procedure. In this case reduction from 7 to 0 points was reached after surgery and only the results of the QLQ-C30 from before and the day after the splanchnicectomy could be analyzed. The rest of the patients (*n* = 28) completed the 12-month follow-up period. None of the patients reported procedure-related pain, which could be diagnosed as intercostal neuralgia requiring treatment. The change in NRS-assessed pain severity is shown in Fig. 1. Twenty-four patients have experienced immediate and significant pain relief postoperatively (NRS 8.26 ± 1.08 vs. 1.85 ± 1.69, *p* < 0.05), and the NRS score remained at a relatively similar level for the rest of the follow-up (postoperative NRS 1.85 ± 1.69 vs. 1.37 ± 1.77 at 12 months, *p* > 0.05). Of the 28 subjects whose complete data were available at 12 months, 10 had completely discontinued analgesics, 16 had significantly reduced the dose of pain medication, and 2 patients treated with neurolysis of the celiac plexus had stayed on the same dose of pain medication. Of the 25 patients in whom bilateral splanchnicectomy was technically performed and who completed the full observation period, 24 patients (96 %) achieved significant pain reduction at 12 months (NRS 0–3). With respect to the whole study population, the success rate at 1 year was 24 of 30 (80 %).

**Table 1:** Characteristics of the study group

Age (years) [mean (range)]	45.03 (28–61)
Sex (F/M)	5/25
Cause of chronic pancreatitis	
Alcohol	25
Gallstones	5
Previous celiac plexus block (Y/N)	3/27
Technical failure (Y/N)	3/27

**Fig. 2:**
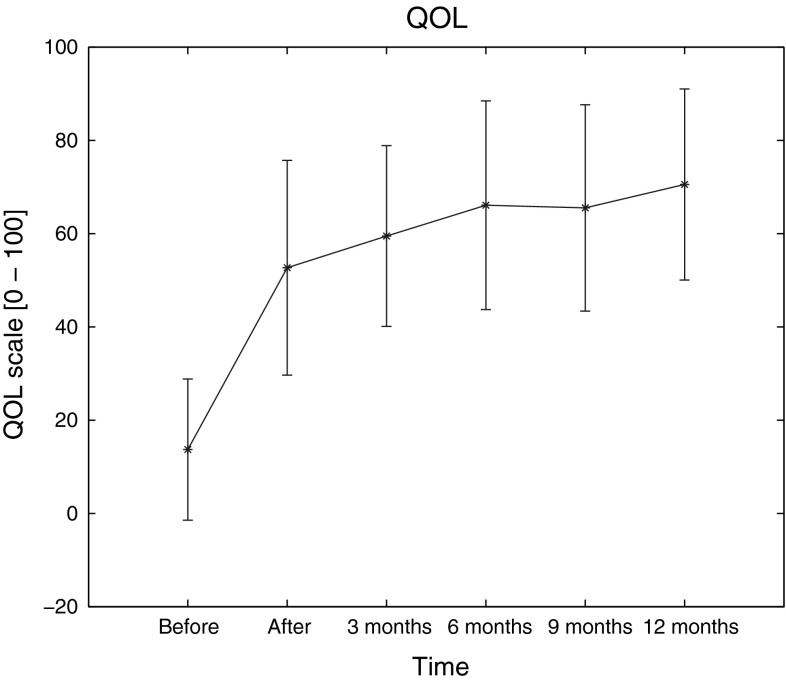
The mean values and standard deviations of the QLQ-C30 results before and after BTS

**Fig. 3:**
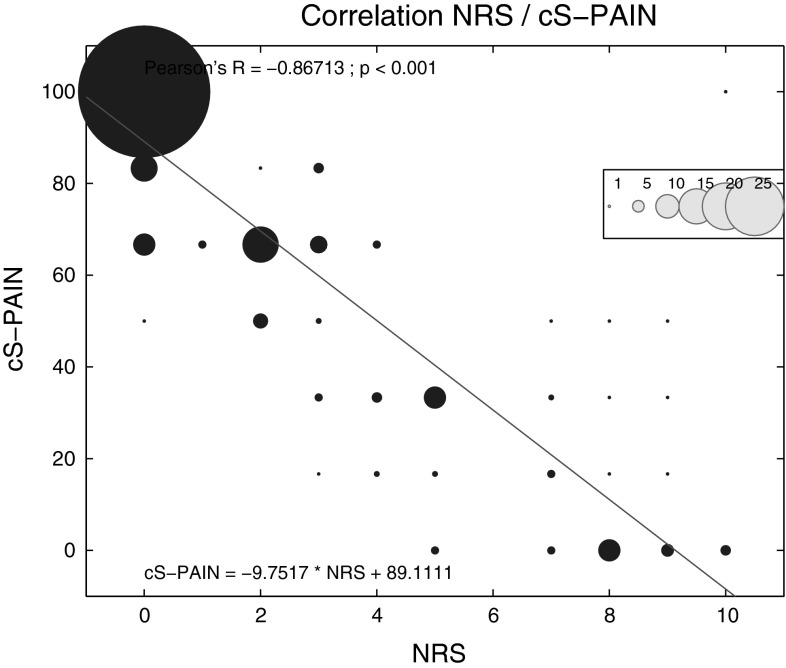
Correlation of QLQ-C30 pain scale results and NRS score. Area of each *circle* refers to the number of the same values on the graph

**Fig. 4:**
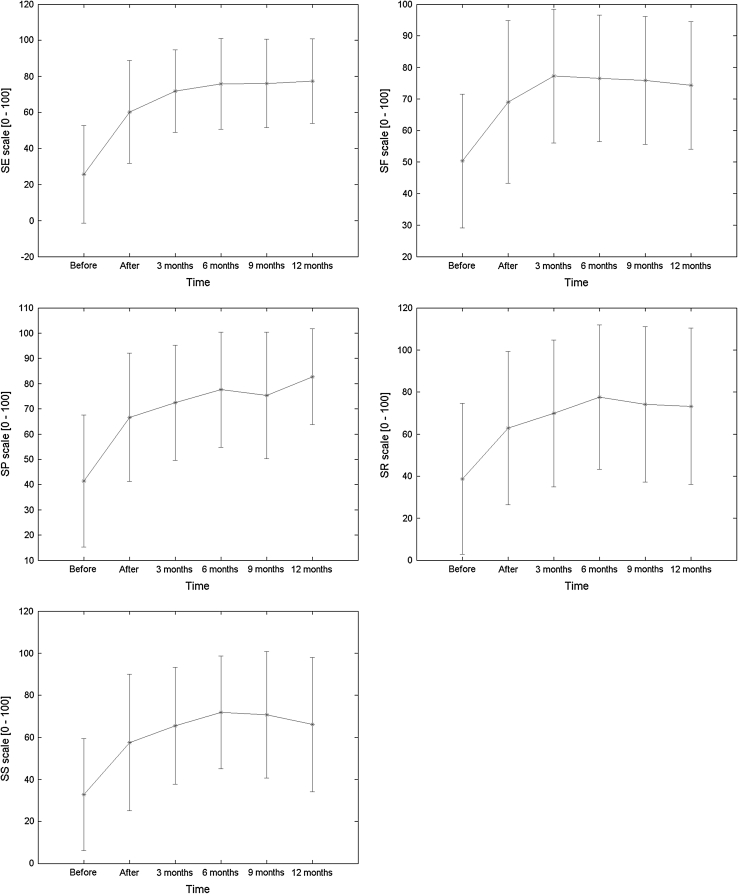
The mean values of the QLQ-C30 functional scales results before and after the BTS. SE—emotional, SF—physical, SP—cognitive, SR—role, SS—social

Improvement in QLQ-C30—measured quality of life is shown in Fig. 2. The mean preprocedure score was 13.7 ± 15.14, which subsequently increased to 52.68 ± 23.01 (*p* > 0.05) postoperatively, and the tendency toward improved QOL continued throughout the follow-up period, resulting in a score of 70.53 ± 20.47 (*p* < 0.001) at 12 months. The correlation between NRS results and the QLQ-C30 pain scale outcomes was calculated in order to confirm good correspondence between the means of pain measure used in the study. As shown in Fig. 3, the correlation coefficient indicates a good relationship between these assessment tools. Additional analysis of the relationship between NRS and the total QOL questionnaire score may lead to the conclusion that the drop in NRS by 1 results in an increase in QOL of 7.77 points (data not shown). The overall outcome of the questionnaire reflects the results of single scales and questions, which are partially shown in Fig. 4. The mean results of all the scales have improved 24 h postoperatively and, apart from diarrhea results, continued to improve over the period of the following 12 months. The cognitive and emotional scales results have shown the greatest increase in the functional assessment group between 1 day after surgery and 12 months of follow-up (66.66 ± 25.45 vs. 82.73 ± 18.96, *p* < 0.05, and 60.21 ± 28.52 vs. 77.38 ± 23.44, *p* < 0.05, respectively). In 12 patients, the improvement in QOL was sufficient enough for them to fully return to work and continue their professional life. Cronbach’s α for most of the multiquestion scales of QLQ-C30 was above 0.7, apart from physical (0.58) and role (0.55) scales, indicating good overall reliability of the questionnaire outcome.

## Discussion

This study has shown the good effect of bilateral splanchnic denervation in controlling chronic pancreatitis-related pain and quality-of-life improvement. The complexity of the pain’s origin and of the routes it follows is the possible reason for the low effectiveness of the therapy aimed solely at pancreatitis [22, 23].

The afferent pain pathway from the pancreas starts in the celiac plexus and runs to the sympathetic trunci on both sides of the spine and on to the central nervous system. Splanchnic nerves arise from the thoracic sympathetic ganglia: the greater splanchnic nerve at T6–T9 and the lesser splanchnic nerve at T10–T11. In some cases the least splanchnic nerve might be identified stemming from the 11th–12th thoracic ganglion [24]. Splanchnic nerves are usually easily visualized during thoracoscopy, although occasionally the parietal pleura on the left side is less transparent due to previous inflammatory processes.

Unlike neurolysis of the celiac plexus, it was indicated that complete transection of the splanchnic nerves within the thoracic cavity was capable of providing long-lasting or even permanent pain relief in chronic pancreatitis. The least invasive technique of denervation is thoracoscopic splanchnicectomy (TS). The first TS was performed by Wittmoser [25]. In 1993, Worsey [26] performed the first TS for pain control in a pancreatic cancer patient. The technique used in present study—bilateral thoracoscopic splanchnicectomy in a prone-positioned patient—was first described by Cuschieri [20]. The prone position allows for bilateral resection of the nerves without changing the patient’s position during the procedure, while a wide incision of the pleura from the 5th to the 12th intercostal space assures the transection of all nerve-bearing tissue.

Debilitating pain in chronic pancreatitis is difficult to manage not only because of the complex nature of the disease but also because it usually occurs in a troublesome group of patients, in whom compliance and quality-of-life assessment may prove to be more challenging than usual. In this study, variation in pain intensity and QOL was successfully measured over 12 months in 28 (93.3 %) cases. Possible immediate analgesic effectiveness of BTS is reported to be as high as 80 %, with minimal risk of complications (morbidity rate 10 %) [12, 21, 27, 28]. Since postoperative QOL and NRS scores greatly improved when compared to preprocedure scores, our immediate results are similar to those from other centers, reporting an average initial success rate of 80 %. Howard et al. [19], in a study of similar design, reported that BTS may be more effective in patients who had no surgical or endoscopic pain-related procedures done earlier. Their results with respect to patients who had no prior pancreatic pain-directed invasive interventions were similar to ours, although in the present study all the subjects had the same duration of follow-up reported, which makes a marked difference in the size of the study population, in our favor. On the other hand, our results are more favorable than those of Hammond et al. [29], where 65 % of patients experienced some reduction in NRS-assessed pain postoperatively, with a substantial number of patients in that study treated invasively beforehand. Although the data on possible factors predicting appropriate pain relief after BTS are limited, the results of a long-term follow-up of 75 cases reported by Buscher et al. [30] suggest that to date there are no identifiable factors that may be considered helpful in predicting the efficacy of BTS for long-term CP pain management, while their 12-month success rate in this regard was 52 %. Although the lack of a unified definition of successful pain relief might be producing some confusion while discussing the results of BTS studies, the above data may indicate that despite its sometimes questionable efficacy, further studies could yield some interesting results, especially on identifying the patients who are most likely to benefit from this procedure. The QOL of our patients improved almost immediately after the procedure, although the overall change did not reach statistical significance. The QOL scores continued to increase over time, thus becoming significant. It is worth noting that the level of NRS-assessed pain throughout the follow-up period did not noticeably change from the day after the procedure, while QLQ-C30 results improved. This supports the general opinion that pain is the most prominent symptom of CP in most cases, and once controlled, other life qualities tend to become more recognized and appreciated. The psychological aspects of the procedure itself might be of some value as well. It is a surgical intervention and as such gives the impression that something substantial is finally being done to battle the most disturbing symptom of the disease, and at the same time it is minor enough to permit the rapid recovery and prompt return to everyday life. Although the placebo effect seems unlikely, considering the sustained pain relief achieved, it is worth mentioning.

The present study summarizes the results of a 1-year follow-up of patients who underwent BTS in our center over 10 years. The follow-up continues and its results will hopefully add to the discussion on the long-term efficacy of this relatively minor and feasible procedure for attenuating intractable pain in chronic pancreatitis. It seems that the important task for the future is to identify possible factors predictive of outcome that would guide the clinicians in the decision-making process when it comes to matching the right patient with the right treatment.
